# Low thyroid hormone receptor alpha-2 (THRα-2) tumor expression is associated with unfavorable tumor characteristics and high breast cancer mortality

**DOI:** 10.1186/s13058-021-01496-7

**Published:** 2021-12-20

**Authors:** Malte Sandsveden, Signe Borgquist, Ann H. Rosendahl, Jonas Manjer

**Affiliations:** 1 Department of Surgery, Skåne University Hospital Malmö, Lund University, Malmö, Sweden; 2 Department of Oncology, Aarhus University Hospital, Aarhus University, Aarhus, Denmark; 3 Department of Clinical Sciences Lund, Oncology, Lund University, Skåne University Hospital, Lund, Sweden

**Keywords:** Breast cancer, Thyroid hormone receptor, Mortality, Tumor characteristics

## Abstract

**Background:**

The active thyroid hormone triiodothyronine (T3) has been found to have an estrogen-like effect on breast cancer cells. Thyroid hormone receptor alpha-2 (THRα-2) acts as an antagonist for triiodothyronine (T3) signaling, and a low expression has been associated with unfavorable tumor characteristics and a higher mortality in breast cancer. However, the evidence are not conclusive. The present study evaluates tumor-specific THRα-2 expression in invasive breast cancers and its association with tumor characteristics and long-term mortality in a large population.

**Method:**

The Malmö Diet and Cancer Study (MDCS), a population-based cohort in Sweden that included 17,035 women from 1991 to 1996, was used. Women diagnosed with breast cancer during 1991–2010 were eligible for inclusion. A tissue micro array was constructed from stored tumor material and stained for THRα-2 using immunohistochemistry. Tumors from 654 patients were scored regarding the intensity and the fraction of cells stained, then dichotomized into low or high expression. Date and cause of death were collected up until 2018-12-31. Tumor- and patient characteristics were available from the MDCS. Missing data was imputed using chained equations. Logistic regression was used to calculate odds ratios (ORs) with 95% confidence intervals (CIs) for low vs high expression of THRα-2 related to specific tumor factors. Mortality was evaluated with Kaplan–Meier curves and Cox regression, rendering hazard ratios (HRs). Analyses were also stratified for estrogen receptor (ER) status.

**Results:**

We found strong evidence of an association between low THRα-2 and unfavorable tumor characteristics, including estrogen receptor negativity: OR 4.04 (95% CI 2.28–7.15) and tumor size > 20–50 mm: OR 2.20 (95% CI 1.39–3.49). We found evidence of increased breast cancer-specific mortality for women with low THRα-2, HR 1.38 (95% CI 0.96–1.99), which remained after adjusting for age at diagnosis, HR 1.48 (95% CI 1.03–2.14), but not after adjusting for relevant prognostic factors, HR 0.98 (95% CI 0.66–1.45). THRα-2 expression in ER-negative tumors had an inverse correlation with overall mortality, HR 0.27 (95% CI 0.11–0.65).

**Conclusion:**

Low tumor-specific THRα-2 expression was in this study associated with prognostically unfavorable tumor characteristics and a higher mortality in breast cancer, but not independent from other prognostic factors.

**Supplementary Information:**

The online version contains supplementary material available at 10.1186/s13058-021-01496-7.

## Introduction

Breast cancer is since 2020 the cancer with the highest incidence worldwide and is also causing the most amount of cancer related deaths among women [1]. Breast cancer is a heterogeneous disease and the prognosis is widely different depending on the tumor characteristics. While tumor size and metastatic spread are of the utmost importance for prognosis, so are tumor grade, proliferation and expression of estrogen- (ER), progesterone- (PgR) and HER2-receptors, and these clinicopathological features guide therapy decision-making [2, 3]. Established treatment options exist for ER- and HER2-positive tumors, while there are limited options regarding triple-negative tumors [4]. There is a clinical need for new therapeutic targets as well as possible prognostic or treatment predictive markers.

Thyroid hormone levels and thyroid function have been associated with breast cancer development and prognosis, but research has not been conclusive. Some studies suggest that hyperthyroidism is a risk factor for developing breast cancer as well as death from breast cancer [5–7]. Other studies have found high thyroid hormone levels to be associated with less aggressive tumor characteristics, and also with improved survival among breast cancer patients [8, 9]. In a systematic review and meta-analysis, the authors conclude that an association exists between hyperthyroidism and breast cancer risk, but that information regarding treatment and potential confounders frequently are missing and the causality thus remains unclear [10]. A possible mechanism involved is that the active thyroid hormone, triiodothyronine (T3), exerts proliferative effects in breast cancer, similar to the effect of estrogen, by binding to thyroid hormone receptors [11].

There are two genes coding for thyroid hormone receptors alpha (THRα) and beta that are then transcribed and spliced into several isoforms with different functions [12]. The T3-binding THRα-1 is the main activating isoform in the alpha gene, while THRα-2 does not have the capacity to bind T3 and instead acts as an antagonist for T3 signaling [12]. The thyroid hormone receptor alpha-2 (THRα-2) has been found to be positively associated with several prognostically favorable tumor factors and a lower mortality in breast cancer [13, 14]. However, these studies have been of retrospective design with small sample sizes, and further research is needed to confirm or dispute these results.

The hypothesis leading up to the present study was that a lower expression of THRα-2 would be associated with prognostically unfavorable tumor characteristics and increased mortality. The aim was to assess whether the expression of THRα-2 in breast tumors is associated with other tumor characteristics, as well as to evaluate any effect on survival.

## Material and method

The present study was designed as a cohort follow-up regarding survival of women with breast cancer within the Malmö Diet and Cancer Study (MDCS), and also as an assessment of their tumor-specific expression of THRα-2. The main endpoint was breast cancer-specific mortality, comparing women with a low intra-tumor expression of THRα-2 with those with a high expression. Breast cancer-specific death was defined as a death where breast cancer was registered as the cause of death, or a contributing factor to it. Secondary endpoints were overall mortality and the association of THRα-2 with other tumor characteristics.

### Study population

The cohort utilized in the present study, the MDCS, consisted of 28,098 individuals (17,035 women) born in the period 1923–1950 and living in the Swedish city of Malmö during the years of recruitment, 1991–1996 [15]. The MDCS enrolled 43% of the eligible women and includes data collected at baseline (blood samples, questionnaires, dietary interviews and more) and data collected later for specific projects (e.g. breast cancer tumor characteristics) [15]. Information regarding breast cancer diagnosis was collected from the Swedish Cancer Register and linked to study participants using their Swedish personal identity number.

**Fig. 1:**
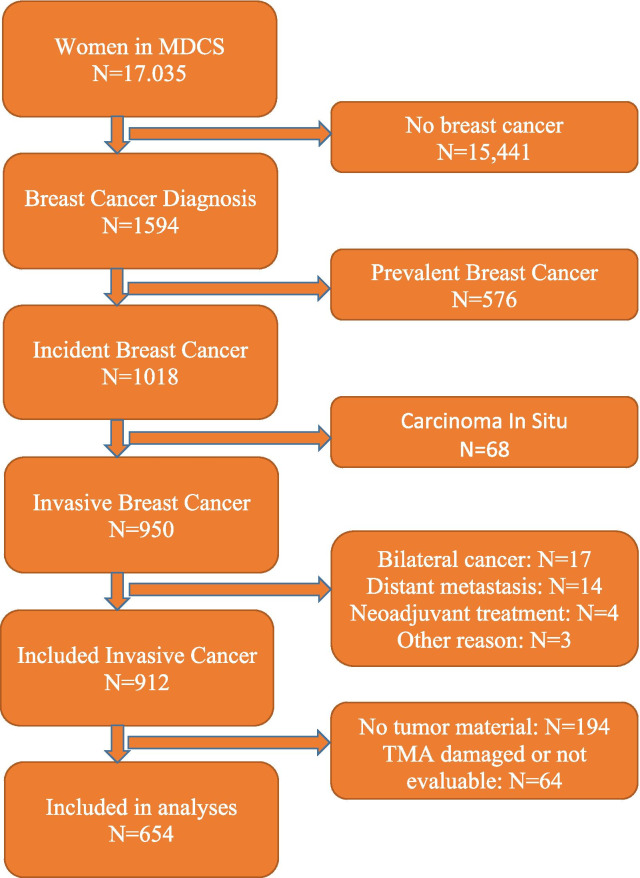
Inclusion and exclusion flowchart

Inclusion and exclusion regarding the present study population is visualized in Fig. 1. All women from the MDCS cohort with a first breast cancer during 1991 to 2010 were eligible for inclusion (*n* = 1018), excluding those with prevalent breast cancer cases at baseline (*n* = 576). Women with carcinoma in situ only were excluded (*n* = 68). Due to difficulty in assessing histological data correctly, women with bilateral breast cancer (*n* = 17), neoadjuvant treatment before surgery (*n* = 4) and distant metastasis at diagnosis (*n* = 14) were also excluded, as well as one woman who refused treatment for four years, one woman who was diagnosed postmortem and one who died before surgery [16, 17]. Out of the remaining eligible 912 women with invasive unilateral breast cancers, 194 had no available tumor material, and for another 64 women their THRα-2 expression could not be assessed because of damaged cores in the tissue micro array (TMA), there were too few tumor cells in the tissue cores, or only in situ carcinoma existed in the cores, leaving a total of 654 women with invasive breast cancer and evaluable tumor tissue that were included in the present study.

### TMA preparation and immunohistochemical staining

The TMAs were created from breast tumors diagnosed in the MDCS up until 2010. Two separate core biopsies of 1 mm were taken from each stored tumor from areas with invasive breast cancer, marked on H&E slides [17]. Each TMA was cut into 3–4 μm sections and transferred to glass slides, dried at room temperature and baked at 60 °C for 1 h. Deparaffinization and antigen retrieval was performed in PT-LINK (Dako/Agilent Denmark A/S). Immunohistochemical staining of THRα-2 receptor was performed with Autostainer Plus using THRA monoclonal antibody (Thermo Fischer Scientific MA1-4676 at 1:100 for 30 min at room temperature) with EnVision Flex, high pH (Agilent K801021-2) and hematoxylin counterstaining.

### THRα-2 evaluation

**Fig. 2:**
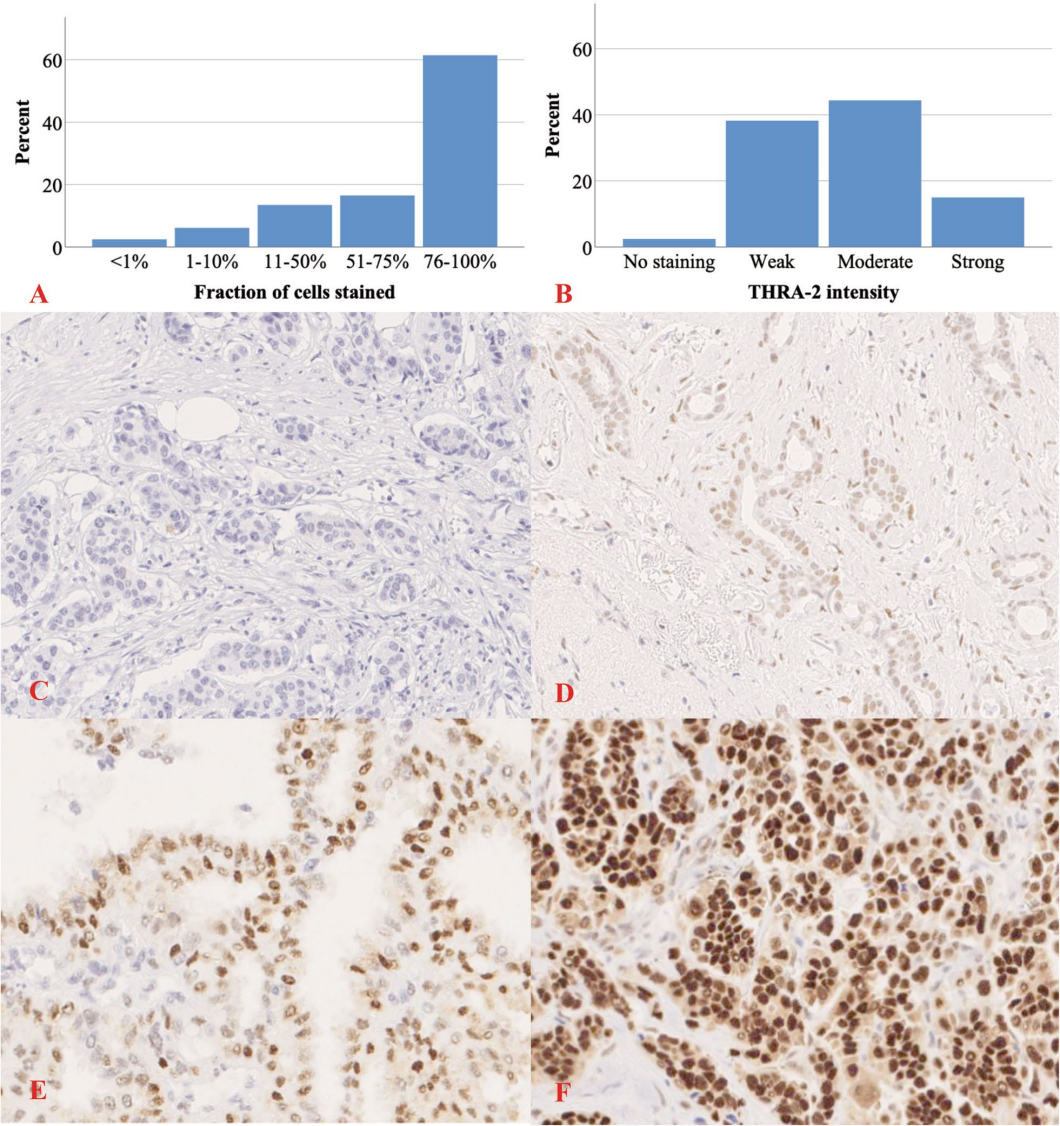
THRα-2 staining patterns and example pictures. **A**, **B** Distribution of fraction and intensity of THRα-2 staining among included women. **C** Tumor with no THRα-2 staining. **D** Tumor with weak THRα-2 staining of > 75% of cells. **E** Tumor with moderate THRα-2 staining of 51–75% of cells. **F** Tumor with strong THRα-2 staining of > 75% of cells.

The cores belonging to the same tumor were evaluated and scored together to obtain a single score for each patient. A minimum of 20 malignant cells was needed for a valid evaluation. The tumors were scored in a semi-quantitative fashion regarding the intensity of nuclear staining: no staining (0), weak (+ 1), moderate (+ 2), strong (+ 3); and fraction of positive cells stained: < 1% (0), 1–10% (+ 1), 11–50% (+ 2), 51–75% (+ 3), 76–100% (+ 4); see Fig. 2 for examples and distribution. The THRα-2 evaluation was performed in two independent readings by author MS (M.D., resident in surgery) who was blinded to patient and tumor characteristics. If the score of a tumor differed between the separate readings in any of the parameters, they were evaluated a third time. If MS could not conclude a score in this reading, author AR (Ph.D., senior researcher) was consulted until consensus was reached. In one unclear case, a senior pathologist was consulted and that tumor was subsequently excluded due to a benign histological diagnosis in the TMA. The concordance was 76.8% (502 pairs) between the first two readings, while in 152 pairs (23.2%) there were conflicting assessments for at least one variable (fraction or intensity). In 41 cases (6.3%), the categorization changed between low and high THRα-2-expression from the first reading to the final dichotomous THRα-2 variable. The evaluation was performed on scanned sections using the digital pathology tool PathXL from Koninklijke Philips N.V. (PHILIPS) (http://www.pathxl.com/).

### Tumor characteristics

Data collection regarding tumor characteristics was performed in three separate periods depending on the date of diagnosis, 1991–2004, 2005–2007 and 2008–2010. In tumors diagnosed in the period 1991–2004, information regarding histological grade, Ki67, HER2-, estrogen receptor (ER)- and progesterone receptor (PgR) status, was collected from a histological re-evaluation as previously described [18]. In accordance with the Swedish clinical guidelines, the threshold of ER and PgR positivity was > 10%. For tumors diagnosed from 2005 onwards, information from medical records was used except regarding HER2- and Ki67 during the period 2005–2007; Ki67 data was collected from TMA and HER2 from national records [19]. Information on tumor size and axillary lymph node involvement (ALNI) was collected from medical records in all periods. The Ki67 data was categorized into low, intermediate or high proliferation, with 1/3 of the tumors in each category separately for the three periods and then combined into one variable.

### Missing values

Missing values in the included 654 individuals were explored in the dataset and evaluated as missing at random. In total, 68.0% of the included women had complete information for all variables. Most missing values were found for the Ki67 variable, 22.2%, while only 0.5% of values were missing regarding tumor size. The missing values were handled by multiple imputation using chained equations (also known as fully conditional specification). This is considered to be a good model for handling missing data when dealing with different variable types including binary, ordered and unordered categorical variables [20]. In total, 25 new datasets including 654 individuals were imputed, using 10 iterations for each dataset. In the final imputation model, the following variables were included: Log(t) (a logarithm to the base 10 of time from diagnosis to endpoint/censoring), breast cancer-specific death, fraction, intensity, age at diagnosis, tumor size (categorical: ≤ 10 mm, 11–20 mm, 21–50 mm > 50 mm), lymph node status, ER-, PgR and HER2- receptor status, Ki67, histological grade and a categorical variable for which time period each women was diagnosed during (1991–2004, 2005–2007 or 2008–2010). For analyses, pooled results of the imputed data were used. The differences between the pooled imputed values and the original data are presented in Additional file 1: Table S1. As a sensitivity analysis, a separate imputation model was created based on the variables in the logistic regression model, excluding mortality data.

### Surrogate intrinsic subtypes

A surrogate intrinsic subtype variable was created from histological parameters as described previously [21]. Four categories were used; Luminal A-like (ER+, HER2- and; grade 1, or; grade 2 and low Ki67, or; grade 2, intermediate Ki67 and PgR+), Luminal B-like (ER+, HER2- and; grade 3, or; grade 2 and high Ki67, or; grade 2, intermediate Ki67 and PgR-), HER2+ (HER2+ regardless of other characteristics) and triple-negative breast cancer (TNBC) (ER-, PgR- and HER2-). The surrogate intrinsic subtypes were constructed from the pooled imputed values.

### Mortality data

The Swedish Cause of Death Register was used to retrieve information on date and cause of death. Information was linked to the MDCS using the Swedish personal identity number. When breast cancer was a causative or a contributory factor to the death, it was classified as a breast cancer-specific death. Time at risk started at date of breast cancer diagnosis and was thus possible from January 1, 1991 up until December 31, 2018 which was the date for end of follow-up.

### Statistical analyses

SPSS v 26 was used for statistical analyses. The tumor-specific expression of THRα-2 was evaluated by multiplying fraction (0–4) and intensity (0–3). The data was dichotomized with the SPSS function *visual binning*; 0–7 was defined as low (*n* = 309, 47%) and 8–12 as high (*n* = 345, 53%). For sensitivity analyses, the data was also categorized in tertiles where 0–4 was defined as low (*n* = 273, 42%), 5–8 as intermediate (*n* = 284, 43%) and 9–12 as high (*n* = 97, 15%).

Odds ratios (ORs) for having a low vs high expression of THRα-2 dependent on the different tumor characteristics were calculated with logistic regression, separately for all tumor characteristics. The same imputed dataset as in the survival analyses was used. Sensitivity analyses with an alternative imputation model based on the logistic regression model were performed to explore if there were any differences compared in results compared to the original imputation model.

Kaplan–Meier curves were used for initial visual assessment of the proportional hazard assumption and mortality differences between women with high vs low THRα-2 tumor expression in the dichotomized model as well as for the tertiles. Due to signs of assumption violation in the late period of the follow-up, log rank test was performed both for the complete period and for 0–15 years of follow-up. Hazard ratios (HRs) were calculated in the dichotomized model with Cox regression, both for overall and breast cancer-specific mortality. The analyses were made in different models: crude, age-adjusted and in a multivariate model adjusted for relevant clinical prognostic factors; tumor size (categorical: ≤ 10 mm, 11–20 mm, 21–50 mm > 50 mm), ALNI, surrogate intrinsic subtype and age at diagnosis. The analyses were performed for the complete follow-up period and limited to 0–15 years of follow-up, both with imputed data as well as for complete cases only.

Furthermore, separate analyses adjusted for the individual tumor characteristics included in the multivariate model were performed and Freedman’s % was calculated to evaluate the contribution of the specific factors. An approach proposed by Freedman et al. [22] to examine whether the effect of an exposure (THRα-2) is reduced when adjusting for an intermediate endpoint (tumor characteristics). The calculation 100*(1-*a*/*b*) defined Freedman’s %, *a* as the logarithm of the adjusted HR and *b* as the logarithm of the unadjusted HR. Additional Cox regression analyses were also performed with the data stratified for ER-status and surrogate intrinsic subtype. Interaction by these factors was tested by including an interaction term in the original regression model.

## Results

Descriptive data regarding included and excluded women is presented in Table 1. The mean age at diagnosis was 65.5 years for the included women. For the 64 women with non-evaluable TMA, the mean age was 62.6 years. Compared with the included women, a larger proportion of the excluded women were diagnosed in the early period. The excluded women were more likely to have smaller tumors and no ALNI, and also more likely to have missing information regarding surrogate intrinsic subtype, tumor size and ALNI. There were no differences in the proportions of the subtypes between the original and the imputed dataset. Included women were followed up to 27 years. Median follow-up time was 12.2 years and the interquartile range 7.7 years.

**Table 1:** Descriptive statistics of included and excluded women

	Included (N = 654)		TMA not	Missing TMA-	Excluded for other
	Imputed	Original	evaluable (*N* = 64)	material (*N* = 194)	reasons (*N* = 106)
Mean age at diagnosis^a^ (SD)	65.5	65.5 (8.0)	62.6 (8.1)	65.9 (8.3)	64.5 (9.1)
BMI (SD)	25.7	25.7 (4.2)	25.2 (4.2)	25.5 (3.7)	25.7 (4.7)
Year diagnosed					
1991–2004	58.1	58.1	67.2	66.0	67.0
2005–2007	22.0	22.0	26.6	15.5	16.0
2008–2010	19.9	19.9	6.3	18.6	17.0
Surrogate intrinsic					
Luminal A-like	55.5	54.5	57.1	62.2	53.3
Subtype					
Luminal B-like	25.3	25.0	25.7	25.6	26.7
HER2+	9.6	10.5	8.6	9.8	13.3
Triple-negative	9.6	10.0	8.6	2.4	6.7
Missing		21.7	45.3	57.7	85.8
Tumor size (mm)					
≤ 10	19.8	19.8	49.2	39.7	36.8
11–20	48.7	48.7	33.3	40.8	28.9
21–50	28.2	28.3	17.5	16.7	25.0
> 50	3.2	3.2	0.0	2.9	9.2
Missing		0.5	1.6	10.3	28.3
Axillary lymph nodes					
No	65.8	64.9	80.4	77.3	77.5
Yes	34.2	35.1	19.6	22.7	22.5
Missing		4.6	12.5	27.3	62.3

Data is presented as valid column % and missing data is presented in total column %. Missing not presented if no missing values

^a^Mean age presented in years

In Additional file 1: Table S2a and S2b, descriptive data regarding different treatment modalities in relation to cause of death and THRα-2 is presented. In total, 397 women were still alive, 254 had died (119 had breast cancer-specific death), and three women had unknown mortality status due to emigration. Among the patients who died of breast cancer, 29.3% had been treated with chemotherapy, whereas only 8% of patients who died of other causes had received chemotherapy. Endocrine treatment was used in 60.4% of those alive, 64.1% of those who died of breast cancer and 57.9% of those who died of other causes. Women with low expression of THRα-2 were more likely to receive chemotherapy (23.6%) and mastectomy (48.3%), compared to women with high THRα-2 expression (11.7% and 38.3% respectively).

Women with low THRα-2 tumor expression were more likely to have tumors with other subtypes than Luminal A-like compared to women with high expression, for Luminal B-like: OR 1.99 (95% CI 1.34–2.97), HER2+: OR 2.42 (95% CI 1.38–4.27) and TNBC: OR 5.10 (95% CI 2.70–9.65). A tendency toward a higher proportion of Luminal A-like tumors with increasing THRα-2 intensity and fraction was observed. Compared with women with high THRα-2 tumor expression, women with low THRα-2 tumor expression were more likely to have ER- and PgR negative tumors, HER2-positive tumors, higher Ki67 and histological grade, as well as larger tumors, and were more likely to have ALNI (Table 2). In the sensitivity analysis using an imputation model based on the logistic regression, similar results were seen (data not shown).

**Table 2:** Tumor characteristics and THRα-2 expression

	THRα-2 fraction					THRα-2 intensity				Combined*		
	< 1%	1–10%	11–50%	51–75%	76–100%	No staining	Weak	Moderate	Strong	Low	High	OR (95% CI)
Surrogate												
Luminal A-like	21.2	30.1	40.1	46.6	65.1	21.2	44.3	62.4	69.1	43.7	66.0	1
Intrinsic subtypes												
Luminal B-like	35.3	35.2	30.5	29.0	21.7	35.3	27.8	23.1	23.8	29.0	22.0	1.99 (1.34–2.97)
HER2+	20.0	13.7	12.2	10.7	7.9	20.0	11.0	10.7	1.2	12.0	7.5	2.42 (1.38–4.27)
Triple-negative	23.6	20.9	17.3	13.6	5.2	23.6	16.9	3.9	5.9	15.3	4.5	5.10 (2.70–9.65)
ER												
> 10%	65.5	71.6	80.5	85.1	93.1	65.5	79.3	94.9	94.1	81.0	94.5	1
≤ 10%	34.5	28.4	19.5	14.9	6.9	34.5	20.7	5.1	5.9	19.0	5.5	4.04 (2.28–7.15)
PgR												
> 10%	6.8	24.4	28.6	54.5	69.4	6.8	29.8	70.3	96.2	35.8	76.3	1
≤ 10%	93.2	75.6	71.4	45.5	30.6	93.2	70.2	29.7	3.8	64.2	23.7	5.77 (4.00–8.33)
HER2												
Negative	80.1	86.3	87.9	89.3	92.1	80.1	89.0	89.4	98.8	88.1	92.6	1
Positive	19.9	13.7	12.1	10.7	7.9	19.9	11.0	10.6	1.2	11.9	7.4	1.69 (0.98–2.92)
Ki67**												
Low	24.5	31.1	36.5	34.1	44.8	24.5	40.7	43.4	34.5	38.8	42.2	1
Intermediate	39.2	30.2	30.1	32.9	31.1	39.2	27.1	31.0	42.0	29.5	33.0	0.97 (0.65–1.47)
High	36.3	38.7	33.5	33.0	24.1	36.3	32.2	25.6	23.5	31.7	24.8	1.46 (1.00–2.12)
Grade**												
Grade I	12.5	7.5	17.5	16.7	30.2	12.5	17.1	32.7	20.6	17.7	30.4	**1**
Grade II	31.3	40.0	45.1	45.4	50.9	31.3	46.6	45.2	63.0	45.2	50.6	**1.53 (1.03–2.29)**
Grade III	56.3	52.5	37.4	38.0	18.9	56.3	36.3	22.2	16.4	37.0	19.0	**3.34 (2.13–5.24)**
Tumor size (mm)**												
≤ 10	12.5	15.0	10.2	16.7	23.6	12.5	18.0	20.8	22.7	16.8	22.5	**1**
11–20	12.5	47.5	47.7	49.1	50.4	12.5	46.0	52.1	51.5	43.7	53.2	**1.10 (0.73–1.67)**
21–50	75.0	30.0	39.8	31.5	22.8	75.0	32.0	23.9	23.7	35.6	21.6	**2.20 (1.39–3.49)**
> 50	0.0	7.5	2.3	2.8	3.3	0.0	4.0	3.1	2.1	3.9	2.6	**1.98 (0.78–5.02)**
ALNI												
No	31.3	60.0	63.5	58.7	70.1	31.3	63.8	68.3	69.1	60.6	70.4	1
Yes	68.8	40.0	36.5	41.3	29.9	68.8	36.2	31.7	30.9	39.4	29.6	1.55 (1.11–2.15)

Odds ratios (ORs) and 95% confidence intervals (CI) for logistic regression low vs high. Pooled imputed data. All data presented as column %

*Combined by multiplying fraction (0–4) and intensity (0–3) and then dichotomized (0–7 = Low, 8–12 = High). **Test for *p*-trend was performed, bold figures indicate *p* < 0.05

**Fig. 3:**
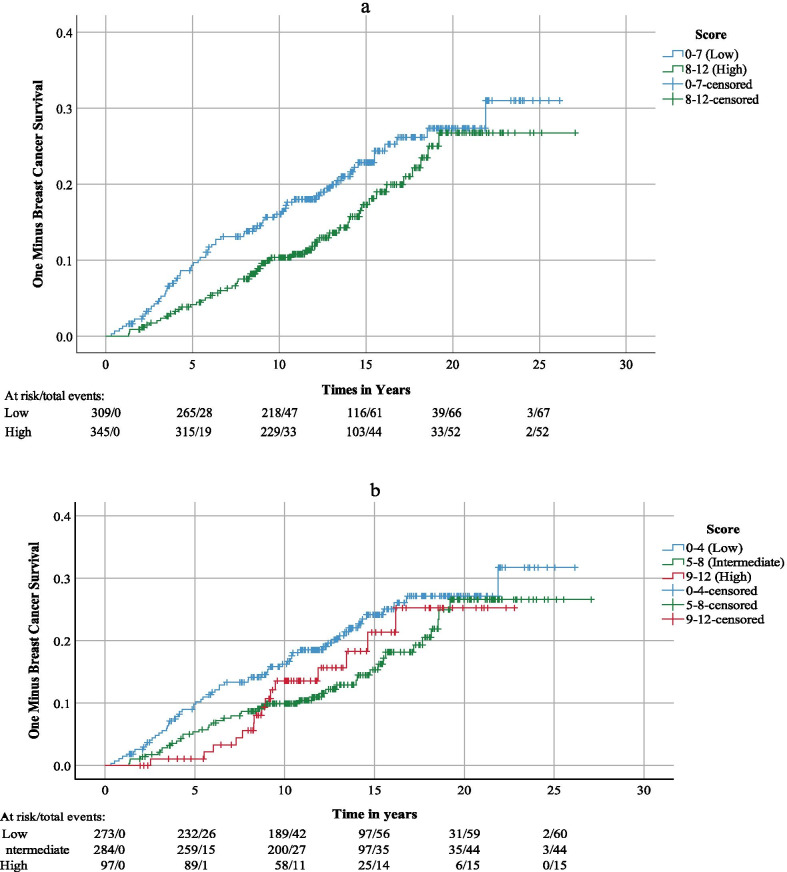
Kaplan–Meier curves for breast cancer specific mortality and THRα-2 tumor expression. In **a** the THRα-2 tumor expression is dichotomized to low and high. In **b** THRα-2, tumor expression is presented in tertiles; low, intermediate and high. For every 5 years of follow-up, the number of individuals at risk and the total number of events up to that point is presented

In the Kaplan–Meier graph, an increased breast cancer-specific mortality was observed in the group with low compared to high THRα-2 expression, up until 18 years of follow-up, at which point the two curves converged (Fig. 3a, log-rank *p* = 0.08). When only looking at the period 0–15 years, the curves between high and low in the dichotomized model were parallel, log rank test *p* < 0.01. Similar results were seen between low and intermediate expression when assessing the THRα-2 expression in tertiles, but there was no evidence of an additional effect from high expression. However, only 97 individuals and 15 events (deaths) were found in the highest tertile and the mortality in that group surpassed that of the intermediate group at eight years and converged with the low group at 16 years (Fig. 3b).

In Table 3, HR for survival and THRα-2 expression is presented. There was a higher breast cancer-specific mortality among women with low, compared with high, THRα-2 tumor expression, HR 1.38 (95% CI 0.96–1.99) for all of follow-up and HR 1.52 (95% CI 1.03–2.24) for the period 0–15 years. When adjusting for only age at diagnosis, similar results were seen, HR 1.48 (95% CI 1.03–2.14). In the fully adjusted model, there was no difference between low and high THRα-2 expression, HR 0.98 (95% CI 0.66–1.45). In the complete case analysis, we found HR 0.81 (95% CI 0.52–1.26). For overall mortality, the unadjusted HR was 1.04 (95% CI 0.82–1.34), adjusted for age at diagnosis: HR 1.22 (95% CI 0.95–1.56), fully adjusted: HR 0.94 (95% CI 0.72–1.23) and complete case analysis: HR 0.89 (95% CI 0.65–1.21). Results for the 15 first years of follow-up were similar to the complete period.

**Table 3:** Hazard ratios (HRs) and 95% confidence intervals (CI) for breast cancer specific and overall mortality per THRα-2 expression

Endpoint	THRα-2	All (*n*)	Deaths (*n*)	HR^crude^ (95% CI)	HR^a^ (95% CI)	HR^b^ (95% CI)	HR^CC^ (95% CI)
Breast cancer specific mortality	Low	309	67	1.38 (0.96–1.99)	1.48 (1.03–2.14)	0.98 (0.66–1.45)	0.81 (0.52–1.26)
All of follow-up	High	345	52	1	1	1	1
	Total	654	119				
Breast cancer specific mortality	Low	309	61	1.52 (1.03–2.24)	1.64 (1.11–2.43)	1.04 (0.68–1.58)	0.86 (0.54–1.39)
0–15 years of follow-up	High	345	44	1	1	1	1
	Total	654	105				
Overall mortality	Low	309	127	1.04 (0.82–1.34)	1.22 (0.95–1.56)	0.94 (0.72–1.23)	0.89 (0.65–1.21)
All of follow-up	High	345	127	1	1	1	1
	Total	654	254				
Overall mortality	Low	309	110	1.16 (0.82–1.34)	1.36 (1.04–1.79)	1.00 (0.75–1.34)	0.94 (0.68–1.32)
0–15 years of follow-up	High	345	102	1	1	1	1
	Total	654	212				

^a^Adjusted for: age at diagnosis

^b^Adjusted for: age at diagnosis, tumor size, axillary lymph node involvement and surrogate intrinsic subtype

^CC^Complete case analysis, adjusted for same as ^b^

When adjusting for one factor at a time, the largest effect on the breast cancer-specific mortality was observed when adjusting for histological grade (HR 1.09, Freedman’s % 72.0), while adjustment for HER2 (HR 1.33, Freedman’s % 7.3) and Ki67 (HR 1.35, Freedman’s % 2.4) only marginally changed the point estimate from the unadjusted analysis (Additional file 1: Table S3). When stratifying the data based on ER-status, a low THRα-2 expression was associated with a higher overall mortality among women with ER+ tumors (HR 1.31 (1.01–1.73) for age adjusted analyses) and a lower overall mortality among ER- (HR 0.44 (0.21–0.94)). Similar point estimates were seen for breast cancer specific mortality and there was evidence of effect modification (*p* < 0.05) by ER-status on THRα-2 expression both regarding breast cancer specific mortality and overall mortality in all analyses (unadjusted, age adjusted and adjusted for age, tumor size and ALNI). No effect modification was seen when stratifying for surrogate intrinsic subtype (Additional file 1: Table S4).

## Discussion

We found evidence of higher breast cancer-specific mortality among women with low expression of tumor-specific THRα-2 compared with those with high expression. The survival difference remained after adjusting for age at diagnosis, but not after adjustment for additional relevant clinical prognostic factors. We found strong associations between low THRα-2 tumor levels and several prognostically unfavorable tumor characteristics including ER negativity, high histological grade and larger tumor size. The results indicate that THRα-2 might be a prognostic marker in breast cancer, but not independent from other prognostic markers.

This is by far the largest study investigating THRα-2 expression and breast cancer, and the present findings are in line with previous research. In recently published research, Zehni et al. [23] found similar to us that THRα-2 expression had a positive association with disease free survival in breast cancer. Ditch et al. [13, 24] found an inverse association between THRα-2 and tumor size, lymph node involvement, histological grade and hormone receptor expression, and an improved disease-free survival among 82 women with higher tumor-specific THRα-2 levels. Jerzak et al. [14] also found evidence of a correlation between higher tumor-specific expression of THRα-2 and prognostically favorable characteristics as well as improved survival among 130 women with invasive breast cancer. However, our results, in contrast to what Jerzak et al. suggest, do not support that THRα-2 is an independent prognostic marker, and the evidence of a survival difference was weaker regarding overall survival. There is also conflicting evidence on outcome in the literature. In a study by Heublein et al. [25], the authors found the expression of THRα-2 to be associated with a reduced five-year survival in BRCA1-associated breast cancers (*n* = 38), but not regarding sporadic cancers (*n* = 86).

Additional findings in the present study were that there were no additional effect regarding high THRα-2 expression compared to intermediate when dividing the data in tertiles, although there were few individuals in the highest tertile. Due to the violation of proportional hazards, the tertiles were not evaluated in Cox regression. Furthermore, our results indicate that ER-status might interact with THRα-2, with a positive association between overall and breast cancer specific survival and THRα-2 status among women with ER-positive tumors and an inverse association among women with ER-negative tumors. The difference between the ER+ and ER- remained after adjusting for age at diagnosis, tumor size and ALNI, which indicates that the effect modification cannot be explained by the general association between THRα-2 and prognostically important characteristics. However, the statistical evidence for a difference was weak, which is partly explained by a low number of cases in the stratified analyses, more so regarding the breast cancer specific mortality, and these findings should be considered exploratory.

Mechanistically, there are several thyroid hormone receptors that mediate the effect of thyroid hormones by gene transcription and the thyroid hormones can have direct effects on cells as well [12]. The activated liganded THRα-1 can both induce and repress gene expression depending on the target gene, while THRα-2 mediates the opposite effect on the gene transcription in the same gene [12]. It has been shown in breast cancer cell lines that T3 can both promote cell proliferation in a similar fashion as estrogen and enhance the effect of estrogen on breast cancer proliferation [11, 26]. It has long been know that T3 and thyroid hormone receptors affect breast epithelial cells. In a review by Muñoz and Bernal [27], the authors summarize that data from animal and in vitro studies support that increased THRα-1 and T3 signaling disrupts the normal phenotype of mammary epithelial cells, while theoretically, THRα-2 should counteract this. In more recent literature, Jerzak et al. [14] found the highest survival among women with low THRα-1 and high THRα-2 expression, supporting that claim. Why some breast cancer express more THRα-2 is not known, but Charalampoudis et al. [28] found that the combined expression of THRα-1 and 2 was lower in invasive ductal breast carcinoma compared to normal breast epithelium, suggesting a loss of expression along with malignant transformation. It is also noteworthy that the association between survival and THRα-2 expression was inversed when looking only at ER-negative tumors. Thyroid hormone receptors have been indicated to modulate the effect of estrogen in previous research [29]. THRα-2 has previously been shown to be positively associated with ER; however in that study the authors found, in contrast to us, that THRα-2 was negatively correlated with tumor size and lymph node spread [13]. Another example of the oncogenic effect of T3 and THRα-1 is that increased signaling has been shown to increase levels of phosphorylated AKT in hepatocellular cancer, and since phosphorylated AKT is overexpressed in several cancers and is also associated with worse prognosis in breast cancer, it could be a possible mechanistic pathway of thyroid hormone receptor signaling in breast cancer as well [30, 31]. Thus, it still remains unclear what pathways THRα-2 might affect in breast cancer cells.

In the present study, we found that only 16 tumors (2.4%) had no expression of THRα-2, while Ditch et al. [13] found that 25% had “negative” THRα-2 staining. In their methodology, they define 0–1 in a multiplicative score ranging from 0 to 12 as negative. If we apply that methodology to our data, 11% would be classified as negative (multiplicative score 0–1). Another reason for this difference could be the use of different antibodies with potentially different sensitivities and specificities. In the study by Jerzak et al. [14], the same antibody as in the present study was used, and by applying the same multiplicative model to that study, around 15% would have a score of 0–1, which is similar to the proportion in our data.

A weakness in this study is that the THRα-2 categories of low/high and low/intermediate/high were set after exploring the dataset and were not pre-defined, which might reduce the generalizability of our results. The reason for applying that methodology was that the field is rather unexplored, and there is no standardized threshold for THRα-2 expression in breast cancer. We believe that our data-driven method is preferable in this situation; however, as more research is presented regarding THRα-2 expression and breast cancer, a standardized and pre-defined histological evaluation and cutoff could minimize bias introduced by researchers in the data. However, a quantitative/semi-quantitative histological evaluation instead of a qualitative assessment is considered superior when assessing other steroid receptors in breast cancer (ER and PgR) and thus was the natural choice of method for us [32].

In the present study, we used TMA to evaluate the THRα-2 expression, and as the receptor is not extensively studied in breast cancer, the tumor-specific heterogeneity is not known. If it resembles the pattern of other steroid receptors as ER and PgR, the concordance of TMA and whole tumor slides could be approximated to around 90% [33].

To optimize the imputation model, White and Royston [34] suggested also including the baseline hazard, in addition to those variables we used. However, that has been questioned, and a model including Log(t) and an event indicator (in the present study death from breast cancer) has been suggested to be a better way to model an imputation suited for Cox regression, and this was our choice of method [35].

Some women with breast cancers were not included in the TMA for various reasons. One reason was that there was not enough tissue available. The present study could therefore have a slight overrepresentation of larger tumors, which is also a predictor of worse prognosis. This could lower the generalizability of the results and is not corrected by just adjusting for tumor size; however, almost 20% of the tumors in our dataset were < 10 mm, indicating that there should not be a major bias in this regard.

## Conclusion

The results of the present study support the hypothesis that a low tumor-specific THRα-2 expression is associated with prognostically unfavorable tumor characteristics and via those associations also a higher mortality in breast cancer. Thus, a low THRα-2 expression acts as a marker of more extensive disease, but it is not an independent predictor of survival in breast cancer. Further research is warranted to evaluate the potential causality, if any, of this association.

## Supplementary Information

**Additional file 1: Table S1.** Pooled imputed values and original values. **Table S2a.** Mortality status and treatment among the 654 women included in survival analyses. **Table S2b.** THRα-2 status and treatment modalities among the 654 women included in survival analyses. **Table S3.** THRα-2 expression, hazard ratios (HRs) with 95% confidence intervals (CI) and Freedman's%, adjusted for a single factor at a time. **Table S4.** Hazard ratios (HRs) and 95% confidence intervals (CI) for overall mortality per THRα-2 expression, stratified for ER-status and surrogate intrinsic subtype.

## Data Availability

The datasets used and/or analyzed during the current study are available from the corresponding author on reasonable request.

## Abbreviations

ALNI: Axillary lymph node involvement
ER: Estrogen receptor
HER2: Human epidermal growth factor receptor 2
HR: Hazard ratio
MDCS: Malmö Diet and Cancer Study
OR: Odds ratio
PgR: Progesterone receptor
T3: Triiodothyronine
THRα: Thyroid hormone receptor alpha
TMA: Tissue micro array
TNBC: Triple-negative breast cancer
