# Supplementary material for: Low thyroid hormone receptor alpha-2 (THRα-2) tumor expression is associated with unfavorable tumor characteristics and high breast cancer mortality
Source: Breast Cancer Res. 2021 Dec 20;23:117. doi: 10.1186/s13058-021-01496-7 (PMC8691018; doi:10.1186/s13058-021-01496-7)
Supplement: Supplementary file 1 — Additional file 1: Table S1. Pooled imputed values and original values. Table S2a. Mortality status and treatment among the 654 women included in survival analyses. Table S2b. THRα-2 status and treatment modalities among the 654 women included in survival analyses. Table S3. THRα-2 expression, hazard ratios (HRs) with 95% confidence intervals (CI) and Freedman's%, adjusted for a single factor at a time. Table S4. Hazard ratios (HRs) and 95% confidence intervals (CI) for overall mortality per THRα-2 expression, stratified for ER-status and surrogate intrinsic subtype. [file 13058_2021_1496_MOESM1_ESM.pdf]

Supplementary Table 1. Pooled imputed values and original values.

		Imputed (N=654)	Original (N=654)
Surrogate intrinsic subtype	Luminal A-like	55.5	54.5
	Luminal B-like	25.3	25.0
	HER2+	9.6	10.5
	Triple-negative	9.6	10.0
	Missing		21.7
Tumor size (mm)	≤10	19.8	19.8
	11-20	48.7	48.7
	21-50	28.2	28.3
	>50	3.2	3.2
	Missing		0.5
Axillary lymph nodes	No	65.8	64.9
	Yes	34.2	35.1
	Missing		4.6
Grade	Grade I	24.4	24.3
	Grade II	48.1	48.1
	Grade III	27.5	27.6
	Missing		1.4
ER	≤10%	11.9	11.5
	>10%	88.1	88.5
	Missing		6.0
PgR	≤10%	42.9	40.7
	>10%	57.1	59.3
	Missing		9.5
HER2	Negative	90.4	90.5
	Positive	9.6	9.5
	Missing		13.1
Ki67	Low	40.6	39.5
	Intermediate	31.3	32.4
	High	28.0	28.1
	Missing		22.2

Data is presented as valid column % and missing data is presented in total column %. Missing not presented if no missing values.

Supplementary Table 2a. Mortality status and treatment among the 654 women included in survival analyses. Data is presented as valid column % and missing data is presented in total column %.

		Alive (N=397)	Breast cancer death (N=119)	Other death (N=135)	Emigrated/Unknown (N=3)
Radiotherapy	No	32.6	36.2	45.6	100.0
	Yes	67.4	63.8	54.4	0.0
	Missing	10.3	2.5	7.4	0.0
Chemotherapy	No	83.1	70.7	92.0	100.0
	Yes	16.9	29.3	8.0	0.0
	Missing	10.6	2.5	7.4	0.0
Hormone therapy	No	39.6	35.9	42.1	33.3
	Yes	60.4	64.1	57.9	66.7
	Missing	2.0	1.7	1.5	0.0
Surgical treatment	Mastectomy	36.0	65.8	44.0	33.3
	Partial Mastectomy	63.8	34.2	56.0	66.7
	Biopsy only	0.3	0.0	0.0	0.0
	Missing	2.0	1.7	7.4	0.0

Supplementary Table 2b. THRα-2 status and treatment modalities among the 654 women included in survival analyses. Data is presented as valid column % and missing data is presented in total column %.

		Low	High
Radiotherapy	No	34.9	37.7
	Yes	65.1	62.3
	Missing	8.1	8.4
Chemotherapy	No	76.4	88.3
	Yes	23.6	11.7
	Missing	8.1	8.7
Hormone therapy	No	39.5	39.3
	Yes	60.5	60.7
	Missing	1.6	2.0
Surgical treatment	Mastectomy	48.3	38.3
	Partial Mastectomy	51.3	61.7
	Biopsy only	0.3	0.0
	Missing	2.9	3.2

Supplementary Table 3. THRα-2 expression, hazard ratios (HR) with 95% confidence intervals (CI) and Freedmans%, adjusted for a single factor at a time.

Endpoint	THRα-2	HR[1] (95% CI)	HR[2] (95% CI)	HR[3] (95% CI)	HR[4] (95% CI)	HR[5] (95% CI)	HR[6] (95% CI)	HR[7] (95% CI)	HR[8] (95% CI)
Breast cancer specific mortality	Low	1.13 (0.77-1.65)	1.25 (0.85-1.82)	1.19 (0.80-1.77)	1.33 (0.92-1.92)	1.35 (0.94-1.95)	1.09 (0.75-1.58)	1.14 (0.79-1.64)	1.28 (0.89-1.84)
	High	1	1	1	1	1	1	1	1
	Freedmans%	60.3%	27.4%	43.4%	7.3%	2.4%	72.0%	57.4%	19.7%
Overall mortality	Low	0.94 (0.72-1.21)	1.00 (0.77-1.29)	1.01 (0.77-1.32)	1.02 (0.79-1.30)	1.04 (0.81-1.33)	0.92 (0.71-1.18)	0.93 (0.72-1.19)	1.01 (0.79-1.30)
	High	1	1	1	1	1	1	1	1

[1]Adjusted for: Surrogate intrinsic subtypes
[2] Adjusted for: ER
[3] Adjusted for: PgR
[4] Adjusted for: HER2
[5] Adjusted for: Ki67
[6] Adjusted for: Histological grade
[7] Adjusted for: Tumor size
[8] Adjusted for: ALNI
Freedmans %: Calculated as $100*(1-\log(HR^{adjusted})/\log(HR^{undadjusted}))$, $HR^{unadjusted}=1.36$.

Supplementary table 4. Hazard ratios (HR) and 95% confidence intervals (CI) for overall mortality per THRα-2 expression, stratified for ER-status and surrogate intrinsic subtype.

Strata	Endpoint	THRα-2	Cases*	Deaths*	HR^{crude} (95% CI)	HR[1] (95% CI)	HR[2] (95% CI)
ER+	Breast cancer specific mortality	Low	250	52	1.43 (0.95-2.14)	1.54 (1.02-2.33)	1.25 (0.83-1.90)
		High	326	45	1	1	1
	Overall mortality	Low	250	104	1.10 (0.43-1.44)	1.31 (1.01-1.73)	1.16 (0.88-1.53)
		High	326	115	1	1	1
ER-	Breast cancer specific mortality	Low	59	15	0.58 (0.23-1.51)	0.55 (0.21-1.41)	0.31 (0.10-0.98)
		High	19	7	1	1	1
	Overall mortality	Low	59	23	0.48 (0.23-1.01)	0.44 (0.21-0.94)	0.27 (0.11-0.65)
		High	19	12	1	1	1
Luminal A-like	Breast cancer specific mortality	Low	135	24	1.23 (0.66-2.28)	1.49 (0.79-2.81)	1.22 (0.64-2.33)
		High	227	19	1	1	1
	Overall mortality	Low	135	44	0.86 (0.59-1.25)	1.15 (0.78-1.70)	1.04 (0.70-1.54)
		High	227	75	1	1	1
Luminal B-like	Breast cancer specific mortality	Low	90	24	1.52 (0.75-3.08)	1.52 (0.75-3.08)	1.40 (0.68-2.86)
		High	76	13	1	1	1
	Overall mortality	Low	90	43	1.28 (0.77-2.11)	1.32 (0.80-2.19)	1.25 (0.75-2.08)
		High	76	28	1	1	1
HER2+	Breast cancer specific mortality	Low	37	10	0.69 (0.27-1.78)	0.69 (0.27-1.80)	0.68 (0.23-2.00)
		High	26	11	1	1	1
	Overall mortality	Low	37	20	0.95 (0.46-1.98)	1.00 (0.48-2.06)	0.99 (0.44-2.26)
		High	26	15	1	1	1
Triple-negative breast cancer	Breast cancer specific mortality	Low	47	13	0.81 (0.26-2.49)	0.79 (0.26-2.42)	0.36 (0.10-1.32)
		High	16	5	1	1	1
	Overall mortality	Low	47	20	0.55 (0.24-1.24)	0.54 (0.24-1.24)	0.28 (0.10-0.74)
		High	16	9	1	1	1

[1] Adjusted for: age at diagnosis.

[2] Adjusted for age at diagnosis, tumor size and axillary lymph node involvement

* The number of individuals and deaths is presented as the mean of all 25 imputed datasets. Since ER-status and the other tumor characteristics that define surrogate intrinsic subtype were imputed if missing, the number of individuals and deaths in a strata can differ between each imputed dataset.
